# Diagnosis and Treatment of Ear Diseases

**Published:** 1880-12

**Authors:** 


Diagnosis and Treatment of Ear Diseases. By Albert H. Buck, M.D., Naval Surgeon to the New York Eye and Ear Infirmary, Instructor in Otology in the College of Physicians and Surgeons in the City of New York. Wm. Wood & Co., New York. 1880.

This, with many other good books, we have received through Mr. Dalston. agent at this place.

It seems that while specialists are abundant and enthusiastic in afflictions of the eye, throat, genito-urinary apparatus, female organs, and so forth, to surfeit the ear, is not blessed with that self-abnegating, ethico-legal horde that conserve, and to a considerable measure, no doubt, preserve the health of the other organs. Aural speciality is not, it seems to us, very popular, and we know of no other way to account for it than the fact that its time has not come yet. Revolving cycles evolve new specialities, and pack upon an already cumbersome literature, theoretical trash. Yet, with all the dross much good gold is mixed, and now and then will appear a practical, common-sense book. From the hasty inspection we have had to give the above work, we are led to commend it as a practical treatise on diseases of the ear, adapted both to the use of practitioner and student.

